# Hydralazine and Enzalutamide: Synergistic Partners against Prostate Cancer

**DOI:** 10.3390/biomedicines9080976

**Published:** 2021-08-07

**Authors:** Nair Lopes, Mariana Brütt Pacheco, Diana Soares-Fernandes, Margareta P. Correia, Vânia Camilo, Rui Henrique, Carmen Jerónimo

**Affiliations:** 1 Cancer Biology and Epigenetics Group, Research Center of IPO Porto (CI-IPOP)/RISE@CI-IPOP (Health Research Network), Portuguese Oncology Institute of Porto (IPO Porto)/Porto Comprehensive Cancer Center (Porto.CCC), Rua Dr. António Bernardino de Almeida, 4200-072 Porto, Portugal; nair.ribeiro.lopes@ipoporto.min-saude.pt (N.L.); mariana.brutt.pacheco@ipoporto.min-saude.pt (M.B.P.); diana.soares.fernandes@ipoporto.min-saude.pt (D.S.-F.); margareta.correia@ipoporto.min-saude.pt (M.P.C.); vania.gomes.camilo@ipoporto.min-saude.pt (V.C.); henrique@ipoporto.min-saude.pt (R.H.); 2 Department of Pathology and Molecular Immunology, School of Medicine and Biomedical Sciences, University of Porto (ICBAS-UP), Rua de Jorge Viterbo Ferreira, 228, 4050-313 Porto, Portugal; 3 Department of Pathology, Portuguese Oncology Institute of Porto (IPO Porto), Rua Dr. António Bernardino de Almeida, 4200-072 Porto, Portugal

**Keywords:** hydralazine, enzalutamide, castration-resistant prostate cancer, epigenetics

## Abstract

Advanced prostate cancers frequently develop resistance to androgen-deprivation therapy with serious implications for patient survival. Considering their importance in this type of neoplasia, epigenetic modifications have drawn attention as alternative treatment strategies. The aim of this study was to assess the antitumoral effects of the combination of hydralazine, a DNA methylation inhibitor, with enzalutamide, an antagonist of the androgen receptor, in prostate cancer cell lines. Several biological parameters, such as cell viability, proliferation, DNA damage, and apoptosis, as well as clonogenic and invasive potential, were evaluated. The individual treatments with hydralazine and enzalutamide exerted growth-inhibitory effects in prostate cancer cells and their combined treatment displayed synergistic effects. The combination of these two drugs was very effective in decreasing malignant features of prostate cancer and may become an alternative therapeutic option for prostate cancer patient management.

## 1. Introduction

Prostate cancer (PCa) is the second most frequently occurring type of cancer and the fifth cause of cancer-related death in men worldwide [1]. The maintenance and progression of PCa depend on androgen signaling [2] and the androgen receptor (AR) is the key player in this process. At advanced stages, therapeutic options mainly focus on androgen-deprivation, leading to AR signaling suppression. Such inhibitory effects are accomplished through AR antagonists and these compounds became standard-of-care in that setting, improving long-term survival [3,4,5].

Enzalutamide belongs to this class of compounds: It is a second-generation AR antagonist, which significantly improved overall survival in a phase III clinical trial [6]. Unlike bicalutamide, enzalutamide displays no agonistic effects. It binds to AR, decreasing its nuclear translocation, diminishing binding to androgen responsive elements, as well as coactivator recruitment [7]. However, PCa cells eventually develop resistance due to aberrant AR signaling [8], rendering the castration-resistant phenotype (CRPC) [9]. For patients with advanced disease the median time to relapse is 16 months [10]. Therefore, alternative therapeutic options are urgently required.

Epigenetic alterations are common events in PCa and may constitute promising therapeutic targets. Among these, DNA methylation is a pivotal event in prostate carcinogenesis [11,12] and many studies have focused on the inhibition of DNA methyltransferases. Hydralazine, commonly used as a vasodilator [13,14], was shown to be a DNA methylation inhibitor [15,16] and several studies have been conducted evaluating its effect in cancer cells [17]. Our group has previously reported the anti-neoplastic effects of hydralazine in PCa cells [18], suggesting that this (repurposed) drug may be of clinical value.

Considering the high mortality rates of CRPC patients and the role of epigenetic modifications in this neoplasia, we hypothesized that the combination of hydralazine with the standard-of-care drug enzalutamide could, at least partially, overcome the resistance that PCa develops to therapy and, eventually, increase patient survival. In order to test this hypothesis, we evaluated the combination of hydralazine with enzalutamide in PCa cells and assessed several phenotypic parameters, including proliferation, apoptosis, invasion, the colony forming ability, and DNA damage.

## 2. Materials and Methods

### 2.1. Cell Culture and Drug Treatments

DU-145, LNCaP, PC-3, PNT1a, and RWPE-1 cell lines were available in our laboratory, whereas C4-2 cells were kindly provided by Prof. Lucia Altucci (University of Campania Luigi Vanvitelli, Italy). Prostate cell lines were all kept in an incubator at 37 °C and 5% CO_2_. DU-145 cells were grown in MEM (PAN-Biotech, Aidenbach, Germany), whereas LNCaP, PC-3, C4-2, PNT1a, and RWPE-1 cells were maintained in RPMI 1640 (PAN-Biotech, Aidenbach, Germany). All cell culture media were supplemented with 10% fetal bovine serum (Biochrom, Berlin, Germany) and 1% penicillin/streptomycin (GRiSP, Porto, Portugal). All prostate cancer cell lines were routinely tested for *Mycoplasma* spp. contamination using two primers: GPO1: ACTCCTACGGGAGGCAGCAGTA and MGSO: TGCAC-CATGTGTCACTCTGTTAACCTC (Sigma–Aldrich/Merck, Darmstadt, Germany).

Hydralazine was obtained from Tokyo Company Industry (TCI, Tokyo, Japan) and dissolved in double-distilled water (B. Braun, Melsungen, Germany), while bicalutamide 10 nM and MDV3100 (enzalutamide) 10 nM solutions (both already dissolved in DMSO) were purchased from APExBIO (Houston, TX, USA). As treatment controls, double-distilled water (B. Braun) was used for hydralazine and DMSO (Sigma–Aldrich/Merck, Darmstadt, Germany) was used for bicalutamide and enzalutamide. The treatments were performed for 72 h: every 24 h the culture medium was discarded and fresh medium containing the respective drug treatment was added.

### 2.2. Cell Viability Assay

The MTT (3-(4, 5dimethylthiazol-2-yl)-2, 5-diphenyl-tetrazolium bromide) assay was used to ascertain cell viability and assays were performed at two different time points: before the first treatment and 24 h after the last treatment. In order to test the individual effect of hydralazine, enzalutamide, and bicalutamide, prostate cells were seeded in 96-well plates and allowed to adhere overnight. Next, cells were treated with different drug concentrations (5–100 μM) and treatments were performed for 72 h. For each viability assay, 0.5 mg/mL of MTT dissolved in culture medium was added to the cells followed by 3 h of incubation at 37 °C and 5% CO_2_ in the dark. DMSO (dimethyl sulfoxide) was used to dissolve formazan crystals and absorbance was measured in a microplate reader (FLUOstar^®^ Omega, BMG Labtech, Ortenberg, Germany) at 540 nm.

### 2.3. Drug Matrix Development and Synergy Assessment

PCa cells (5 × 10^3^) were seeded in 96-well plates and allowed to adhere overnight. In the following day, they were submitted to various combinations of drug treatments in a 6 × 6 matrix. Daily treatments were performed for 72 h. The drug concentrations used were based on the EC_50_ (half-maximal effective concentration) values determined by the cell viability assays. Control wells were included containing both untreated and vehicle-treated cells. Cell viability was evaluated by MTT before the first treatment and 24 h after the final treatment. The CompuSyn software [19] was used to calculate the drug combination index (CI), under the assumption that a value lower than 1 indicates synergistic effects, a value of 1 denotes an additive effect, and a value higher than 1 implies antagonistic effects.

### 2.4. Apoptosis Assay

PCa cells (2 × 10^5^ for all cell lines) were seeded in flasks and in the following day submitted to treatment with the combination of hydralazine and enzalutamide at the concentrations previously calculated. Daily treatments were performed for 72 h. Identification of apoptotic PCa cells was accomplished with the FITC Annexin V Apoptosis Detection kit with 7-AAD (Biolegend, San Diego, CA, USA) in accordance with the manufacturer’s protocol. Data acquisition was performed by flow cytometry using a FACS Canto^TM^ II Cell Analyzer (BD Biosciences, Franklin Lakes, NJ, USA) and analysis was carried out using FlowJo^TM^ software (BD Biosciences, Franklin Lakes, NJ, USA).

### 2.5. Proliferation Assay

PCa cells were seeded in 96-well plates at 5 × 10^3^ per well and in the following day submitted to treatment with the combination of hydralazine and enzalutamide at the concentrations previously calculated. Daily treatments were performed for 72 h and assays were performed at two different time points: before the first treatment (0 h time point) and 24 h after the last treatment. Cell proliferation was evaluated using the Cell Proliferation ELISA, BrdU (colorimetric) kit (Roche/Sigma–Aldrich/Merck, Darmstadt, Germany) in accordance with the manufacturer’s guidelines. Absorbance was measured in a microplate reader (FLUOstar^®^ Omega, BMG Labtech, Ortenberg, Germany) at 450 nm with background subtraction at 690 nm. All the absorbance values were normalized for the initial time point (0 h) and the vehicle condition.

### 2.6. Cell Cycle Profile Analysis

PCa cells (2 × 10^5^ for all cell lines) were seeded in flasks and in the following day submitted to treatment with the combination of hydralazine and enzalutamide at the concentrations previously calculated. Daily treatments were performed for 72 h. Cell cycle profiles were assessed using the Phase-Flow^TM^ BrdU kit (Biolegend, San Diego, CA, USA) in accordance with the manufacturer’s protocol. Data acquisition was performed by flow cytometry using a FACS Canto^TM^ II Cell Analyzer (BD Biosciences, Franklin Lakes, NJ, USA) and analysis was carried out using FlowJo^TM^ software (BD Biosciences, Franklin Lakes, NJ, USA).

### 2.7. Clonogenic Assay

PCa cells (1 × 10^3^ PC-3 cells per well, 2 × 10^3^ for C4-2 and 3 × 10^3^ for LNCaP cells) were seeded in 6-well plates and on the following day treated with the combination of hydralazine and enzalutamide at the concentrations previously calculated. Daily treatments were performed for 72 h. Upon colony formation (after 5 additional days), cell colonies were washed with PBS (phosphate buffered saline), fixed with methanol (Supelco/Sigma–Aldrich/Merck, Darmstadt, Germany) for 10 min and washed again with PBS. Cell colonies were incubated with Hemacolor solution 2 (Sigma–Aldrich/Merck, Darmstadt, Germany) for 1 min, followed by washing with PBS. Next, Hemacolor solution 3 (Sigma–Aldrich/Merck, Darmstadt, Germany) was added for 1 min and washed with PBS. Finally, cells were washed with running water for 2 min and left to dry overnight. The survival fraction (SF) was calculated considering the plating efficiency (PE) of the control. PE = number of colonies counted on control ÷ number of cells plated × 100 and SF = number of colonies counted ÷ (cells plated × [PE ÷ 100]).

### 2.8. Single Cell Gel Electrophoresis (Comet Assay)

PCa cells (2 × 10^5^ for all cell lines) were seeded in flasks and the following day submitted to treatment with the combination of hydralazine and enzalutamide at the concentrations previously calculated. Daily treatments were performed for 72 h. DNA damage was assessed using the protocol described elsewhere [19]. Representative pictures were taken on a microscope (model IX51, Olympus, Tokyo, Japan) and cells were analyzed using the ImageJ software OpenComet Plugin, which measured the tail moment: tail % DNA × means of head × tail distance. At least 500 cells were included per condition.

### 2.9. Cell Invasion Assay

PCa cells (2 × 10^5^ for all cell lines) were seeded in flasks and the following day submitted to treatment with the combination of hydralazine and enzalutamide at the concentrations previously calculated. The treatments were performed for 72 h. One day after the last treatment, cells were harvested and seeded in serum-free medium in Matrigel^®^ invasion chambers (Corning, Corning, NY, USA) and incubated at 37 °C and 5% CO_2_ for an additional 24 h. In the following day the chambers were washed with PBS, the cells in the upper part of the chamber were removed and the ones present on the lower part of the chamber incubated with paraformaldehyde 4% (ChemCruz/Santa Cruz Biotechnology, Dallas, TX, USA) for 2 min, followed by washing with PBS. Next, they were fixed with methanol (Supelco/Sigma–Aldrich/Merck, Darmstadt, Germany) for 20 min and washed again with PBS. The membrane was stained with Crystal Violet (Active Motif, Carlsbad, CA, USA) for 10 min, washed with PBS and the whole insert was photographed under a stereomicroscope (model S2 × 16, Olympus, Tokyo, Japan). The invasive cells were counted using the ImageJ software Cell Counter Plugin.

### 2.10. Statistical Analysis

Statistical analyses were performed using GraphPad Prism 7. Differences between conditions were evaluated using the Kruskal–Wallis test, with Dunn’s correction. In all analyses performed, *p* values lower than 0.05 were considered statistically significant: * *p*  <  0.05; ** *p*  <  0.01; *** *p*  <  0.001; **** *p*  <  0.0001. All the data are presented as mean ± standard deviation for each group and are representative of at least three independent experiments.

## 3. Results

### 3.1. Hydralazine, Enzalutamide, and Bicalutamide Inhibit Prostate Cancer Cell Growth

**Figure 1:**
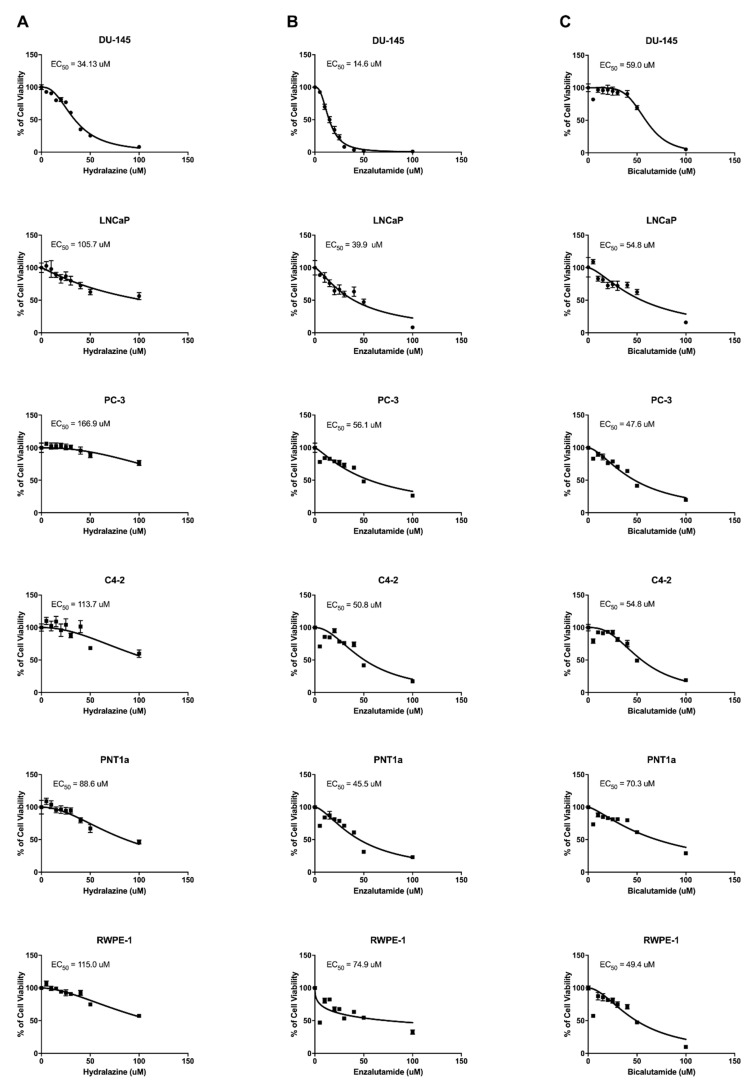
Dose–response curves of prostate cell lines upon hydralazine (**A**), enzalutamide (**B**), and bicalutamide (**C**) treatment. EC_50_ values after 72 h of treatment are indicated in each graph.

The cytotoxic effect of each drug was assessed by calculating the EC_50_ values of hydralazine, enzalutamide, and bicalutamide in DU-145, LNCaP, PC-3, and C4-2 cells following 72 h of drug treatment. Hydralazine EC_50_ values were 34.1, 105.7, 166.9, and 113.7 for DU-145, LNCaP, PC-3, and C4-2, respectively (Figure 1). Enzalutamide exhibited EC_50_ values of 14.6, 39.9, 56.1, and 50.8 in DU-145, LNCaP, PC-3, and C4-2, respectively, whereas the values determined for bicalutamide were 59.0 for DU-145, 54.8 for LNCaP, 47.6 for PC-3, and 54.8 for C4-2. Overall, DU-145 cells were shown to be the most sensitive cells to hydralazine and enzalutamide treatments, but were the most resistant to the treatment with bicalutamide. Conversely, PC-3 cells were the least responsive cells to hydralazine and enzalutamide and the ones most sensitive to the bicalutamide treatment. The EC_50_ values of hydralazine, enzalutamide, and bicalutamide were also determined in the normal prostate cell lines PNT1a and RWPE-1 and the values obtained were 88.6 and 115.0 for hydralazine, 45.5 and 74.9 for enzalutamide, and 70.3 and 49.4 for bicalutamide, respectively. The PNT1a cell line exhibited a much higher bicalutamide EC_50_ value in comparison with the PCa cells, whereas the same trend was observed for RWPE-1 cells regarding enzalutamide, demonstrating a specific effect of these drugs in the tumor context. We did not observe any tendency regarding the hydralazine EC_50_ values in normal prostate cells. An overview of all EC_50_ values is displayed in Table 1.

### 3.2. Drug Combinations Exhibit Synergistic Effects on Growth Inhibition of Prostate Cancer Cell Lines

**Figure 2:**
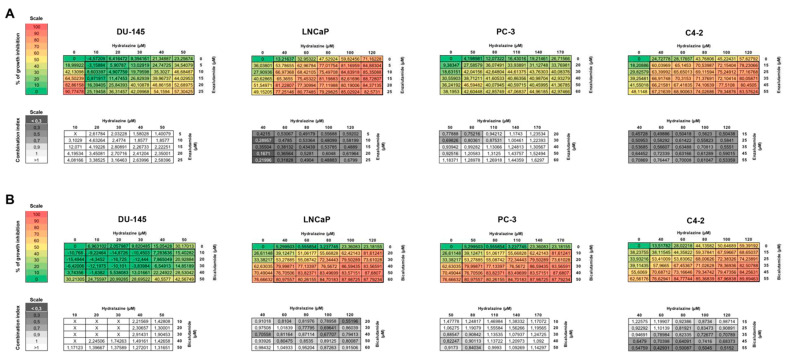
Combination matrices of hydralazine and enzalutamide (**A**) or bicalutamide (**B**) in PCa (prostate cancer) cell lines. The CompuSyn software was used to calculate the drug combination index, with a value lower than 1 indicating synergistic effects, a value of 1 denoting an additive effect, and a value higher than 1 implying antagonistic effects. (X—impossible to calculate).

Next, 6 × 6 drug matrices were built using the EC_50_ values determined for each drug individually as a reference and the combination indexes (CIs) were calculated using the CompuSyn software. The combination of hydralazine and enzalutamide in DU-145 cells showed only antagonistic effects, with CI values above 1 and a similar result was observed for the combination of hydralazine and bicalutamide in the same PCa cell line (Figure 2). In contrast, hydralazine and enzalutamide displayed synergistic effects in LNCaP cells, hydralazine 40 μM/enzalutamide 20 μM being the pair with the lowest CI (0.167). The combination of hydralazine 120 μM/bicalutamide 20 μM yielded the best CI (0.552) in LNCaP prostate cancer cells. For PC-3 cells, the combinations hydralazine 50 μM/enzalutamide 30 μM and hydralazine 50 μM/bicalutamide 40 μM were the most synergistic, with CI values of 0.698 and 0.822, respectively. Regarding C4-2 cells, the combination hydralazine 40 μM/enzalutamide 15 μM and hydralazine 60 μM/bicalutamide 55 μM yielded the best CI: 0.457 and 0.429, respectively. Collectively, with the exception of C4-2 cells, the combination of hydralazine and enzalutamide displayed a stronger synergistic effect than the combination of hydralazine and bicalutamide. Hence, subsequent functional studies were performed using only the combination of hydralazine and enzalutamide (Table 1).

### 3.3. The Combination of Hydralazine and Enzalutamide Attenuate the Malignant Phenotype of PCa Cells

**Figure 3:**
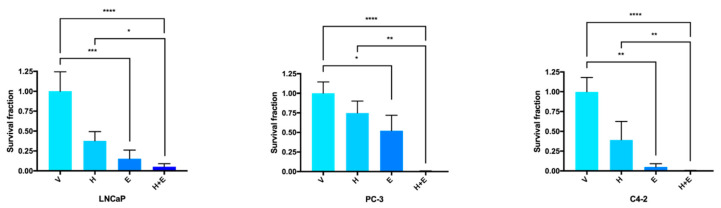
The combined treatment of hydralazine and enzalutamide reduced the colony formation ability in PCa (prostate cancer) cells. Values were normalized to the vehicle condition. (V—vehicle, H—hydralazine, E—enzalutamide, H+E—combination of hydralazine and enzalutamide).

Since hydralazine and enzalutamide inhibited PCa cell growth, the clonogenic assay was performed in order to confirm the effect of these drugs, alone or in combination, as cytotoxic agents. All the PCa cells displayed an impaired ability to form colonies upon hydralazine or enzalutamide treatment alone compared to the respective control cells, with enzalutamide causing a significantly lower survival fraction than hydralazine (Figure 3). When both drugs were combined, there was a synergistic effect and the survival fraction was even lower. Significant differences in the survival fraction were also observed between the single treatment with hydralazine and the combination of drugs for all cell lines.

**Figure 4:**
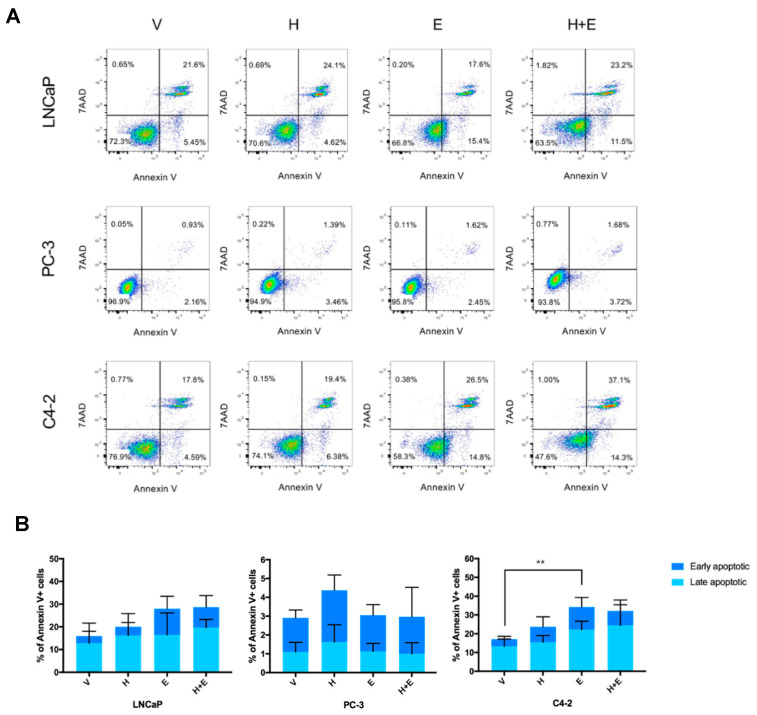
The combination of hydralazine and enzalutamide induced apoptosis in LNCaP and C4-2, but not in PC-3 cells. Representative Annexin V/7AAD dot plots (**A**) and data quantification (**B**). (V—vehicle, H—hydralazine, E—enzalutamide, H+E—combination of hydralazine and enzalutamide).

After confirming the cytotoxic effect of the combination of hydralazine and enzalutamide, it was important to address if the treatment with these drugs induced apoptosis in PCa cell lines. Upon hydralazine treatment there was an increase in the percentage of apoptotic cells in comparison with the control condition for all cell lines (Figure 4A,B). The treatment with enzalutamide also induced an augmented percentage of Annexin V-positive LNCaP and C4-2 cells, with a more pronounced outcome than hydralazine, but showed no effect on PC-3 cells. The combination of both drugs provoked an increment of apoptosis in LNCaP and C4-2 cells compared with the vehicle-treated condition, but did not affect the PC-3 cell line.

**Figure 5:**
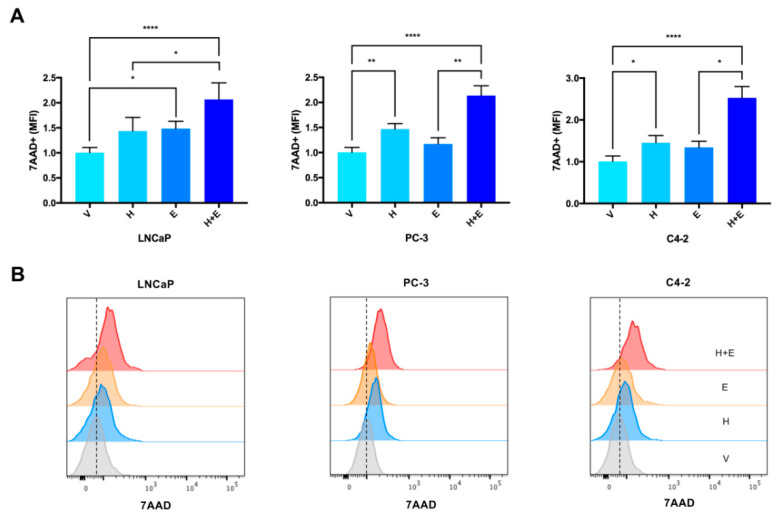
The combined treatment of hydralazine and enzalutamide induced an increase in the amount of 7AAD in PCa (prostate cancer) cells. Values were normalized to the vehicle condition (**A**). Corresponding histograms depicting the amount of 7AAD (**B**). (MFI—mean fluorescence intensity, V—vehicle, H—hydralazine, E—enzalutamide, H+E—combination of hydralazine and enzalutamide).

Although the apoptotic response induced by the combination of hydralazine and enzalutamide in LNCaP and C4-2 cells was similar to the one induced by enzalutamide alone (Figure 4), the number of dead cells (measured by 7AAD) upon combined treatment was higher compared with each drug individually, for all the cell lines tested (Figure 5). In accordance, the combined drug treatment yielded less live cells than any of the drugs individually (Figure S1).

**Figure 6:**
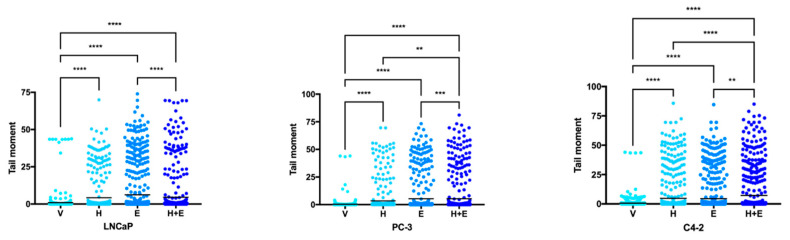
The combined treatment of hydralazine and enzalutamide augmented DNA damage (here represented by the tail moment measurement) in PCa (prostate cancer) cells. The presented data are representative of at least 500 cells analysed. The mean ± standard deviation were as follows: LNCaP V—0.96 ± 5.33; LNCaP H—4.23 ± 10.8; LNCaP E—6.19 ± 13.9; LNCaP H+E—4.53 ± 12.9; PC-3 V—0.36 ± 2.84; PC-3 H—3.36 ± 10.84; PC-3 E—5.35 ± 14.18; PC-3 H+E—5.34 ± 14.67; C4-2 V—0.66 ± 3.31; C4-2 H—4.74 ± 13.33; C4-2 E—4.45 ± 12.46; C4-2 H+E—7.42 ± 16.85 (V—vehicle, H—hydralazine, E—enzalutamide, H+E—combination of hydralazine and enzalutamide).

Our group had previously demonstrated that hydralazine exposure caused DNA damage in PCa cells [18]. Therefore, the comet assay was performed in order to determine whether the combined treatment of hydralazine and enzalutamide might be eliciting the same effect and, consequently, leading to cell death. The treatment with hydralazine and enzalutamide individually induced greater DNA damage (here represented by the tail moment measurement) in comparison with the control condition in all the tested cell lines (Figure 6 and Figure S2). In LNCaP and PC-3 cells, enzalutamide was more efficient than hydralazine in causing DNA damage, whereas in the C4-2 cells both drugs showed a similar effect compared with the vehicle-treated condition. Upon drug combination there was also a significant increase in the tail moment in all PCa cells in comparison with the control. Additional differences were observed between the treatment with enzalutamide and the combined drugs in all cell lines, with the single drug treatment being more effective than the combinatory one in LNCaP and PC-3 cells, and the combination of drugs inducing a stronger effect than enzalutamide alone in C4-2 cells. PC-3 and C4-2 cells also exhibited a significant increase in tail moment upon the combination treatment when compared with the individual hydralazine condition.

**Figure 7:**
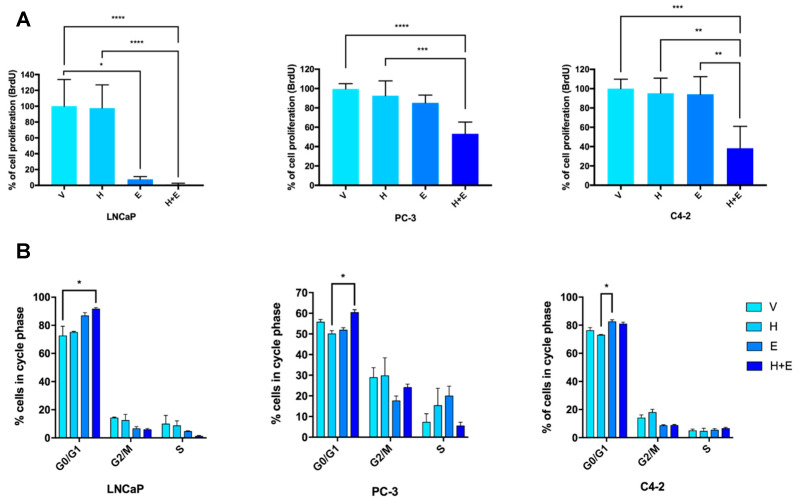
The combination of hydralazine and enzalutamide decreased cell proliferation in PCa (prostate cancer) cells. Percentage of cell proliferation (evaluated by BrdU assay). Values were normalized to the vehicle condition (**A**). Corresponding cell cycle analysis of PCa cells (**B**). (V—vehicle, H—hydralazine, E—enzalutamide, H+E—combination of hydralazine and enzalutamide).

The effect of hydralazine and enzalutamide on cell proliferation was also evaluated, as it might constitute an additional reason for the observed growth inhibition. In comparison with vehicle-treated cells, hydralazine marginally reduced proliferation in PCa cells (Figure 7A). Upon enzalutamide treatment, LNCaP exhibited a significant reduction in cell proliferation, while a similar trend was observed in PC-3 and C4-2 cells. For all the cell lines, the lowest cell proliferation index was obtained with the combination of the two drugs, demonstrating a synergistic effect. Additionally, significant differences were detected between hydralazine and the combination condition for all cell lines, as well as between enzalutamide and the combined treatment for C4-2 cells.

The cell cycle profile analysis depicted the distribution of PCa cells upon drug treatment, thus complementing the obtained cell proliferation data. Overall, an increased percentage of cells in the G0/G1 phase was observed upon the combinatorial treatment for all the tested cell lines, with significant differences between LNCaP cells exposed to the combined treatment and the control, and between PC-3 cells treated with the combined drugs and hydralazine (Figure 7B). In C4-2 cells, enzalutamide significantly upregulated the percentage of G0/G1 cells comparing with hydralazine. Conversely, for all cell lines, the combined exposure to hydralazine and enzalutamide decreased the percentage of cells in the G2/M phase and the same effect was observed for cells in the S phase, except for the PC-3 cell line. Moreover, the percentage of cells in the sub-G1 phase was in accordance with the apoptosis data (Figure S3).

**Figure 8:**
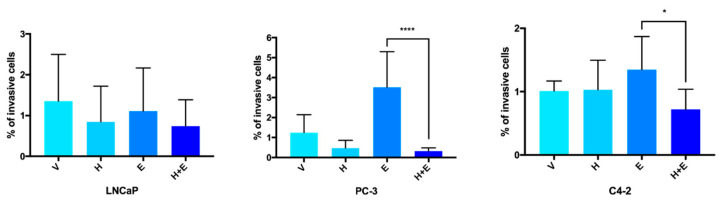
The combined treatment of hydralazine and enzalutamide downregulated the invasion potential of PCa (prostate cancer) cell lines. Values were normalized to the vehicle condition. (V—vehicle, H—hydralazine, E—enzalutamide, H+E—combination of hydralazine and enzalutamide).

The response to hydralazine and enzalutamide was further explored through the evaluation of the effect of the drug treatment, alone or in combination, in PCa cells invasive capacities. A reduction in cell invasion was observed in LNCaP and PC-3 cells upon hydralazine exposure when compared with control cells (Figure 8). However, hydralazine showed no effect on cell invasion in C4-2 cells. The treatment with enzalutamide decreased the percentage of LNCaP invading cells, whereas an increment in cell invasion was observed for PC-3 and C4-2 cells when compared to the vehicle-treated condition. A decline in cell invasion was apparent in all the cell lines upon the combination of hydralazine and enzalutamide, comparing with the control. Furthermore, a significant downregulation of cell invasion was detected comparing the single enzalutamide condition and the combination of drugs for PC-3 and C4-2 cells.

## 4. Discussion

PCa remains a considerable health burden due to high morbidity and mortality rates [1]. Although the majority of tumors is initially responsive to androgen-deprivation therapy (ADT) with drugs such as enzalutamide, they eventually become refractory and highly aggressive, entailing poor survival rates [9,20]. Hence, alternative and effective therapeutic strategies are required to treat these patients and improve disease outcome.

In this study, we explored the synergistic effects of the combination of hydralazine, a repurposed epi-drug, with the standard-of-care ADT drugs enzalutamide and bicalutamide. Interestingly, several reports have evaluated the synergistic effects of combinatory strategies using either hydralazine [21,22,23], enzalutamide [24,25,26] or bicalutamide [27,28,29,30] with other agents as potential anti-neoplastic therapies, but, to the best of our knowledge, this is the first study combining these drugs and assessing their effects in PCa cell lines.

All the cell lines evaluated were sensitive to the growth inhibitory effects of hydralazine, enzalutamide, and bicalutamide. Furthermore, the enzalutamide EC_50_ values were overall lower than those obtained for bicalutamide, for all the cell lines tested, with the exception of PC-3 cells. These results are consistent with the observation that enzalutamide is more efficient than bicalutamide in blocking AR signaling, in part due to its higher binding affinity and the lack of agonistic effects [7].

The hypothesis of combining drugs relies on the existence of synergistic effects, allowing the use of lower doses, precluding side effects, including toxicity, and increasing the time lag until resistance development. This approach is particularly attractive for cancers in which conventional therapies have failed or tumors became refractory [31], such as CRPC. Drug matrices combining hydralazine with either enzalutamide or bicalutamide have shown synergistic effects on both settings for LNCaP, PC-3, and C4-2 cells. Additionally, our results have demonstrated that the synergistic effects of the combination of hydralazine with enzalutamide were more pronounced than those obtained for the combination of hydralazine with bicalutamide and, thus, the functional effects on several biological parameters of PCa cells were only evaluated for the former combination.

Collectively, the functional assays have confirmed the data obtained from the drug matrices, revealing that the combination of hydralazine and enzalutamide induced a stronger biological response than any of the single agents. The drug combination condition was highly effective in preventing colony formation, while individual treatments caused a less pronounced reduction in the clonogenic capacities of PCa cells, a result that is supported by a previous report in which enzalutamide, alone or in combination with a tyrosine kinase inhibitor, was shown to hinder the formation of colonies of LNCaP cells [32].

Additionally, as both hydralazine [33,34] and enzalutamide [35,36,37] were shown to promote an apoptotic response in various tumor models, the effect of their combination was also assessed in PCa cells. An increased apoptotic response was detected upon the combined treatment in LNCaP and C4-2, but not in PC-3 cells. Although the combination condition was unable to promote a greater effect than enzalutamide alone in all cell lines, the combinatorial treatment had already caused cell death, preventing the detection of a large number of apoptotic cells during the Annexin V assay. Indeed, upon 72 h of treatment, the combination of hydralazine and enzalutamide significantly upregulated the number of dead cells and simultaneously yielded the lowest number of live harvested cells in all the PCa cell models. In agreement with this, both hydralazine and enzalutamide have induced a significant upregulation of the tail moment in all the cell lines tested. Also supporting our results, previous reports have shown that hydralazine caused DNA damage in PCa and leukemic T cells [18,34], whereas enzalutamide induced cell cycle arrest [38,39]. Nonetheless, the drug combination condition displayed a more potent response than any single agent.

The combination of hydralazine and enzalutamide also significantly hindered cell proliferation in LNCaP, PC-3, and C4-2 cells, thus contributing to PCa cells growth inhibition. Furthermore, hydralazine and enzalutamide as single agents also affected the PCa cells growth capacity. This corroborates previous results of the single agents in ovarian [40], breast [33], cervical [41,42], and prostate [25,26,32,35,43] cancer. In agreement with other studies [35,38,39], enzalutamide individually increased the percentage of LNCaP and C4-2 cells in the G0/G1 phase, while decreasing the percentage of LNCaP cells in the G2/M and S phase. Contradictory results were observed for hydralazine, where cell cycle arrest in the G0/G1 phase and a reduction in the percentage of cells in the G2/M were described for PC-3 cells [18], whereas we detected no differences. This discrepancy may be attributed to the different drug concentrations used in the different studies.

Moreover, the combinatorial treatment with hydralazine and enzalutamide has diminished the invasive capacities of all the analyzed PCa cells, with a stronger effect than any of the individual drugs. This is an important finding, considering the high mortality rates of prostate cancer due to metastasis development [44]. Regarding the treatment outcome of the single agents, our data confirmed that hydralazine downregulates cell invasion [45], whereas conflicting results were reported regarding enzalutamide, which has been described both as a suppressor [46], as well as a promoter of invasion [47].

Collectively, our results demonstrate that the combination of hydralazine and enzalutamide is able to reduce tumorigenic properties of PCa cells. Importantly, these drugs exhibit synergistic effects, being the combinatorial treatment more effective than the individual drugs in eliciting biological responses, thus raising the possibility of an alternative therapeutic strategy for specific subgroups of PCa patients. In future studies, it would be interesting to use enzalutamide-resistant cell lines and evaluate whether the combination treatment of enzalutamide and hydralazine could overcome enzalutamide resistance, because hydralazine, alone or in combination with valproic acid, was shown to revert resistance to chemotherapy and induce radiosensitization [48,49,50]. Furthermore, it would be important to pinpoint the pathways responsible for the observed combinatorial effects, as well as to validate these results using in vivo models.

## Figures and Tables

**Table 1:** EC_50_ values and selected drug combinations for functional studies in prostate cell lines.

Prostate Cell Lines	Hydralazine (uM)	Enzalutamide (uM)	Bicalutamide (uM)	H+E (uM)
DU-145	34.1	14.6	59.0	NS
LNCaP	105.7	39.9	54.8	40 + 20
PC-3	166.9	56.1	47.6	50 + 30
C4-2	113.7	50.8	54.8	40 + 15
PNT1a	88.6	45.5	70.3	NT
RWPE-1	115.0	74.9	49.4	NT

H+E—combination of hydralazine and enzalutamide, NS—no synergistic effect; NT—not tested.

## Data Availability

The main data supporting the findings of this study are available within the paper and its Supplementary Information.

## Supplementary Materials

The following are available online at https://www.mdpi.com/article/10.3390/biomedicines9080976/s1, Figure S1: The combined treatment of hydralazine and enzalutamide reduced the number of live harvested prostate cancer cells after 72 h of treatment. Values were normalized to the vehicle condition. (V—vehicle, H—hydralazine, E—enzalutamide, H+E—combination of hydralazine and enzalutamide). Figure S2: Representative image of the comet assay using prostate cancer cells. (V—vehicle, H—hydralazine, E—enzalutamide, H+E—combination of hydralazine and enzalutamide; magnification 200x). Figure S3: Percentage of prostate cancer cells in sub-G1 phase after 72 h of treatment with hydralazine and enzalutamide. (V—vehicle, H—hydralazine, E—enzalutamide, H+E—combination of hydralazine and enzalutamide).
