# Supplementary material for: Hydralazine and Enzalutamide: Synergistic Partners against Prostate Cancer
Source: Biomedicines. 2021 Aug 7;9(8):976. doi: 10.3390/biomedicines9080976 (PMC8391120; doi:10.3390/biomedicines9080976)
Supplement: Supplementary file 1 [file biomedicines-09-00976-s001.zip › biomedicines-1257361-supplementary.pdf]

biomedicines

MDPI

Supplementary materials

Hydralazine and Enzalutamide: Synergistic Partners Against Prostate Cancer

Nair Lopes [1], Mariana Brütt Pacheco [1], Diana Soares-Fernandes [1], Margareta P. Correia [1,2], Vânia Camilo [1], Rui Henrique [1,2,3] and Carmen Jerónimo [1,3,*]

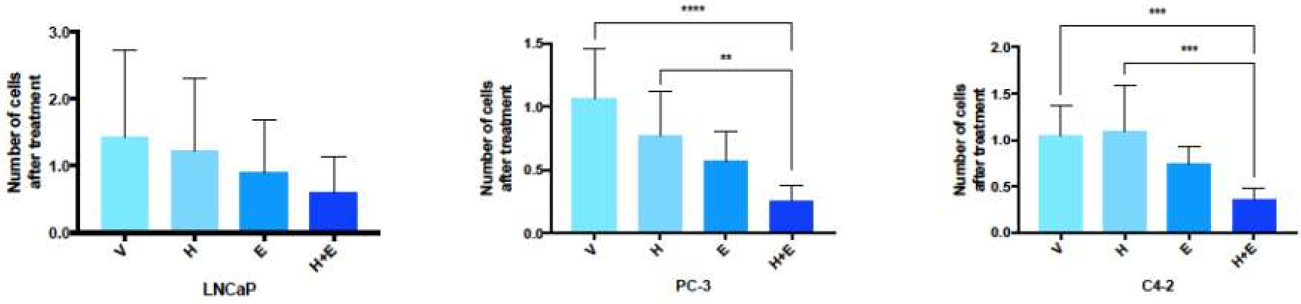

Figure S1. The combined treatment of hydralazine and enzalutamide reduced the number of live harvested prostate cancer cells after 72 hours of treatment. Values were normalised to the vehicle condition. (V–vehicle, H–hydralazine, E–enzalutamide, H+E–combination of hydralazine and enzalutamide)

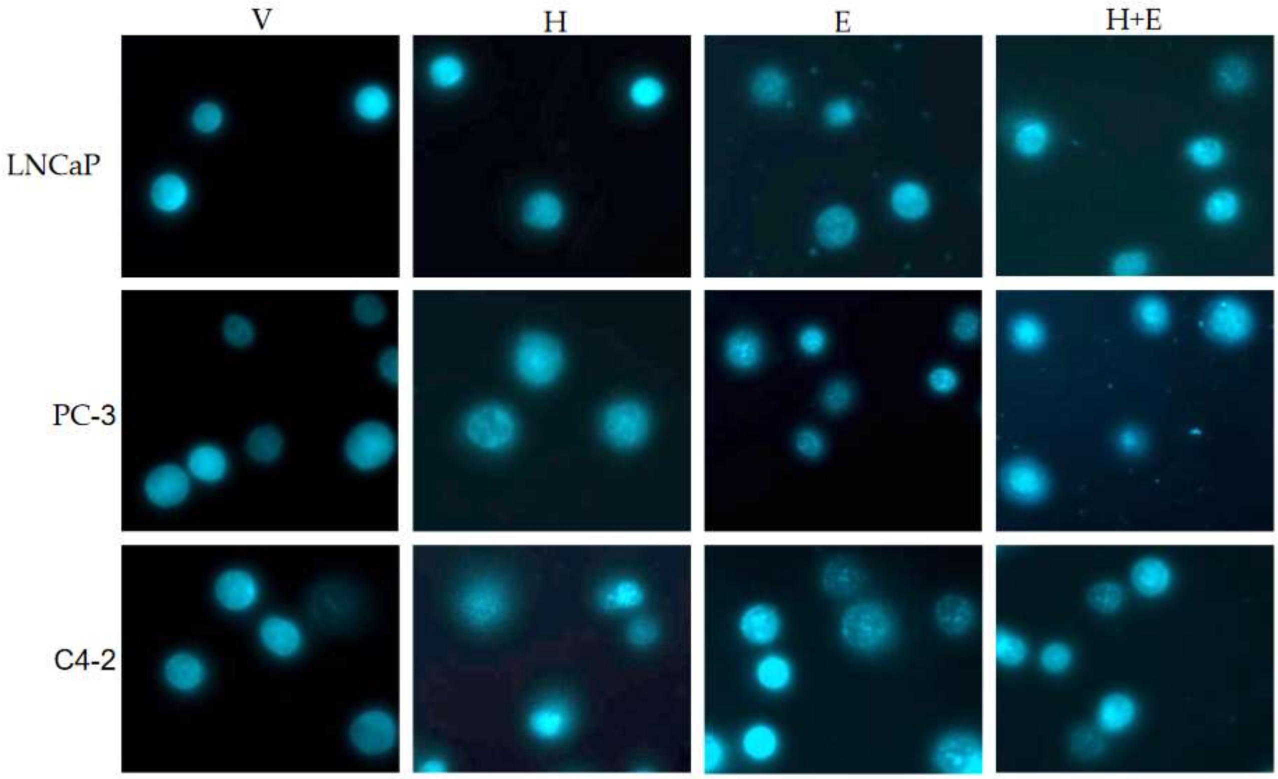

Figure S2. Representative image of the comet assay using prostate cancer cells. (V-vehicle, H–hydralazine, E–enzalutamide, H+E–combination of hydralazine and enzalutamide; magnification 200x).

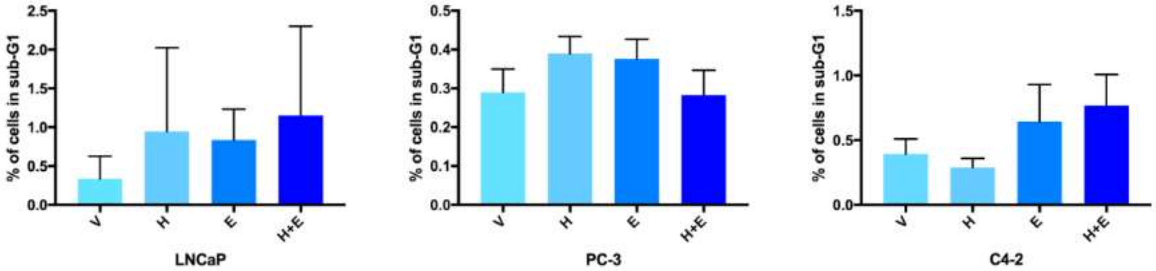

Figure S3. Percentage of prostate cancer cells in sub-G1 phase after 72 h of treatment with hydralazine and enzalutamide. (V–vehicle, H–hydralazine, E–enzalutamide, H+E–combination of hydralazine and enzalutamide).
